# Genetic Analyses of *Elys* Mutations in *Drosophila* Show Maternal-Effect Lethality and Interactions with *Nucleoporin* Genes

**DOI:** 10.1534/g3.118.200361

**Published:** 2018-05-17

**Authors:** Kazuyuki Hirai, Zhuo Wang, Kohei Miura, Takaaki Hayashi, Takeshi Awasaki, Moe Wada, Yoko Keira, Hiroyuki O. Ishikawa, Kyoichi Sawamura

**Affiliations:** * Department of Biology, Kyorin University School of Medicine, Mitaka, Tokyo, 181-8611 Japan; † Graduate School of Life and Environmental Sciences; § Faculty of Life and Environmental Sciences, University of Tsukuba, Tsukuba, Ibaraki, 305-8572 Japan; ‡ Graduate School of Science, Chiba University, Chiba, Chiba, 263-8522 Japan

**Keywords:** nuclear pore complex, maternal-effect lethal, fertilization, interspecific hybrids, centrosome

## Abstract

ELYS determines the subcellular localizations of Nucleoporins (Nups) during interphase and mitosis. We made loss-of-function mutations of *Elys* in *Drosophila melanogaster* and found that ELYS is dispensable for zygotic viability and male fertility but the maternal supply is necessary for embryonic development. Subsequent to fertilization, mitotic progression of the embryos produced by the mutant females is severely disrupted at the first cleavage division, accompanied by irregular behavior of mitotic centrosomes. The *Nup160* introgression from *D. simulans* shows close resemblance to that of the *Elys* mutations, suggesting a common role for those proteins in the first cleavage division. Our genetic experiments indicated critical interactions between ELYS and three Nup107–160 subcomplex components; hemizygotes of either *Nup37*, *Nup96* or *Nup160* were lethal in the genetic background of the *Elys* mutation. Not only *Nup96* and *Nup160* but also *Nup37* of *D. simulans* behave as recessive hybrid incompatibility genes with *D. melanogaster*. An evolutionary analysis indicated positive natural selection in the ELYS-like domain of ELYS. Here we propose that genetic incompatibility between *Elys* and *Nups* may lead to reproductive isolation between *D. melanogaster* and *D. simulans*, although direct evidence is necessary.

The nucleoporins (Nups) consist of ∼30 distinct proteins that constitute the nuclear pore complex (NPC; for recent reviews, see Dickmanns *et al.* 2015; Hurt and Beck 2015; Kabachinski and Schwartz 2015). NPCs are distributed throughout the nuclear envelope and provide the gate for nucleocytoplasmic transport of macromolecules like proteins and RNAs during interphase. They are disassembled and reassembled in open mitosis and have roles in mitosis, such as spindle assembly, kinetochore function, chromosome segregation and possibly centrosome formation (Resendes *et al.* 2008; Güttinger *et al.* 2009).

The Nup107–160 subcomplex, which consists of nine Nups, is the early key player for NPC assembly. ELYS (embryonic large molecule derived from yolk sac), which was originally discovered in mice as a transcription factor (Kimura *et al.* 2002), recruits the NPC to the nuclear envelope, kinetochore and mitotic spindle via the association between ELYS and the Nup107–160 subcomplex (Fernandez and Piano 2006; Galy *et al.* 2006; Franz *et al.* 2007; Gillespie *et al.* 2007; Rasala *et al.* 2006, 2008; Chatel and Fahrenkrog 2011; Clever *et al.* 2012; Bilokapic and Schwartz 2013; Inoue and Zhang 2014; Morchoisne-Bolhy *et al.* 2015; Schwartz *et al.* 2015; Gómez-Saldivar *et al.* 2016).

ELYS is essential for mice; a null mutant is lethal at the early embryonic stage (Okita *et al.* 2004). In contrast, the *Caenorhabditis elegans* homolog, MEL-28 (maternal-effect embryonic-lethal-28), which—as its name suggests—has a required maternal effect and is dispensable for zygotic development (Fernandez *et al.* 2014). Although a BLAST search against the *Drosophila melanogaster* genome suggested that gi:24643345 (= *CG14215*) encodes the ELYS homolog (Rasala *et al.* 2006), no analyses of the gene were undertaken in *Drosophila* (Chen *et al.* 2015). Ilyin *et al.* (2017) recently conducted the immunological staining of ELYS in ovarian somatic cells of *Drosophila*.

Here we disrupted the X-linked *CG14215* (hereafter, *Elys*) of *D. melanogaster* and analyzed the mutant phenotypes. Surprisingly, the *D. melanogaster* mutants exhibited an effect similar to the *C. elegans* mutants; homozygotes (or hemizygotes) were viable and male-fertile but female-sterile (maternal-effect lethal). Sperm penetrated the eggs produced by the mutant females, but the first mitotic division was never completed. This is one of the earliest developmental defects caused by *D. melanogaster* mutations (for the list of the genes, see Loppin *et al.* 2015) and will provide a rare opportunity to analyze *Drosophila* fertilization (Callaini and Riparbelli 1996; Kawamura 2001). In the present report we will describe in detail the developmental defects of the embryos in which maternally supplied ELYS is depleted.

The introgression of the *Nup160* allele from *D. simulans* (*Nup160^sim^*) causes recessive female sterility in the *D. melanogaster* genetic background (Sawamura *et al.* 2010). Females homozygous or hemizygous for *Nup160^sim^* produce eggs capable of sperm entry, but the embryos never develop (Sawamura *et al.* 2004). As this is similar to the maternal-effect phenotype of the *Elys* mutations, we wanted to compare these phenotypes in detail. We also show genetic interaction between *Elys* and the *Nups*, and discuss the possible involvement of ELYS in reproductive isolation between *D. melanogaster* and *D. simulans*.

## Materials and Methods

### Fly strains

For *D. melanogaster* strains used, see FlyBase (Gramates *et al.* 2017; http://flybase.org/). *Int(2D)D+S* carries *D. simulans* introgressions including *Nup160^sim^* (Sawamura *et al.* 2000), and *Df(2L)Nup160M190* is a deficiency that only disrupts *Nup160* (Maehara *et al.* 2012). The *Nup98–96* gene is dicistronic and the *Nup98–96^339^* mutation only disrupts *Nup96* (Presgraves *et al.* 2003).

To eliminate endosymbiotic bacteria (presumably *Wolbachia*) from fly stocks used for embryo immunostaining, we fed flies with medium containing 0.03% tetracycline for one generation (Hoffmann *et al.* 1986). This allowed us to analyze chromosomal DNA exclusively with DAPI staining, but not coexistent bacterial DNA, in the early Drosophila embryo (Lin and Wolfner 1991; Kose and Karr 1995).

### Establishment of Elys mutations

**Figure 1:**
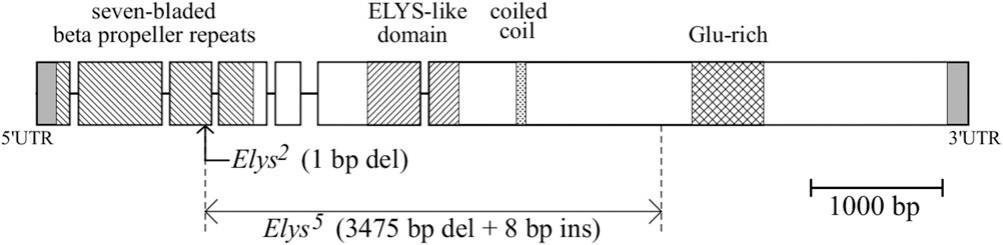
Structure of the *Elys* gene and its mutations. Box, exon; horizontal line, intron. 1–490 aa, seven-bladed beta propeller repeats; 714–922 aa, ELYS-like domain; 1,069–1,092 aa, coiled coil; 1,665–1,847 aa, Glu-rich. There was a 1-bp deletion (1,287T) in *Elys^2^* and a 3,475-bp deletion (1,293–3,512) in *Elys^5^*; 5′-CTC GGT CG-3′ was inserted at the latter site instead.

No *Elys* mutations had been reported in *D. melanogaster*. Generation of *Elys* alleles was carried out with the *CRISPR/Cas9* system described previously (Kondo and Ueda 2013). The guide RNAs (gRNAs) were selected using CRISPR Optimal Target Finder (Gratz *et al.*, 2014; http://tools.flycrispr.molbio.wisc.edu/targetFinder/). To generate a double gRNA construct to target the *Elys* locus, two pairs of oligonucleotides were annealed and cloned into the pBFv-U6.2B vector; one of the pairs of oligonucleotides is 5′-CTT CGC TGC ACT CGG TCT GCT ACA-3′ and 5′-AAA CTG TAG CAG ACC GAG TGC AGC-3′, and the other is 5′-CTT CGG CCA CTG ACT CGT TGC TCG-3′ and 5′-AAA CCG AGC AAC GAG TCA GTG GCC-3′. The *Elys* gRNA vector was injected into embryos of *y^1^ v^1^ P{y^+t7.7^ = nos-phiC31\int.NLS}X*; *P{y^+t7.7^ = CaryP}attP40*. The transgenic U6-*Elys*-gRNA flies were established, and mutations in the *Elys* locus were recovered in offspring from *nos-Cas9* (*y^2^ cho^2^ v^1^*; *attP40{nos-Cas9}/CyO*) and the U6-*Elys*-gRNA flies. *Cas9*-mediated targeted mutagenesis of the *Elys* locus was introduced on the X chromosome of *y^2^ cho^2^ v^1^*. Potential mutations of the *Elys* locus were identified by genomic PCR using the primers 5′-AAG ACG GCC GAA TCC TGA TCT ACG-3′ and 5′-AGA CCA CTA GAC TGC GTT GCT TGC-3′; these primers sandwich the potential deletions (the former is on exon 3 and the latter is on exon 7). Sequencing of the obtained PCR products confirmed mutations of the corresponding genomic region (Figure 1).

### Embryo collection and immunostaining

Well-fed virgin females were mated with wild-type (Oregon-R) males and allowed to lay eggs in short vials containing fly medium on which yeast was seeded. Embryos were collected at 20-min intervals, and the following fixation was completed within an additional 10 min. After dechorionation with 50% bleach for 1.5 min, embryos were washed with water and then fixed and devitellinized by shaking in a mixture of equal volumes of heptane and methanol. Fixed embryos were stored in methanol.

Embryos were rehydrated with PBT (PBS with 0.1% Triton X-100), blocked in PBT and 2% normal goat serum (Vector Laboratories) for 3 hr at room temperature and incubated with primary antibodies in PBT for 24 hr at 4°. We used rat monoclonal anti-Tubulin (YL1/2, 1:300; Abcam) and rabbit anti-Centrosomin (Cnn) (1:3000; Lucas and Raff 2007). Cnn, a component of pericentriolar material crucial for mitotic centrosome assembly (Megraw *et al.* 1999; Vaizel-Ohayon and Schejter 1999; Lucas and Raff 2007), is a mitotic centrosome marker. Embryos were washed in PBT and incubated with secondary antibodies Alexa Fluor 488-conjugated goat anti-rat IgG (1:800; Thermo Fisher Scientific) and Cy3-conjugated AffiniPure goat anti-rabbit IgG (1:800; Jackson ImmunoResearch Laboratories) in PBT overnight at 4°. After an addition of DAPI (final concentration, 2 μg per ml) to stain DNA, incubation was continued for an additional 3 hr at 4°. After extensive washing in PBT, embryos were mounted in Fluoro-KEEPER antifade reagent (Nacalai Tesque). The preparations were imaged as *z*-series acquired at 0.5-µm intervals on a FLUOVIEW FV1000 with a 60×/1.30 Sil UPlanSApo objective (Olympus). Images were then processed as maximum-intensity projections using ImageJ (NIH) and Adobe Photoshop CS6 (Adobe Systems).

To visualize sperm in the eggs, females were crossed with *w*; *dj-GFP/CyO* males, which produce fluorescent sperm tails (*dj*, *don juan*; Santel *et al.* 1997). Egg collection, dechorionation and methanol fixation were performed as described above, followed by replacement of methanol with ethanol. Fixed eggs were stepped gradually into PBT by sequential transfers into PBT containing 75%, 50%, 25% and 0% ethanol and then were stored at 4°. For observation, eggs were incubated in 25% glycerol in PBS, mounted on glass slides with SlowFade Gold antifade reagent (Thermo Fisher Scientific) and then coverslipped by using a small amount of silicone grease (HIVAC-G, Shinetsu Silicone) to avoid egg-rupture.

### Evolutionary analyses of Elys

By using *Elys* of *D. melanogaster* (*CG14215*) as a query, homologs of *D. simulans* (*GD26978*), *D. sechellia* (overlapping *GM22978* and *GM22979*) and *D. yakuba* (*GE15862*) were obtained by a BLAST search (blastn in FlyBase). The sequences were aligned by using Clustal X ver. 2.1 (Larkin *et al.* 2007) and corrected manually. The number of nonsynonymous substitutions per nonsynonymous site (*K_a_*) and the number of synonymous substitutions per synonymous site (*K_s_*) were calculated, and the *K_a_*/*K_s_* ratio test (Li 1993) was conducted by using the kaks function in the seqinR package for the R environment (Charif and Lobry 2007; http://seqinr.r-forge.r-project.org). The *K_a_*/*K_s_* ratio was also calculated within a 180-bp sliding window to increase the sensitivity. PAML (Phylogenetic Analysis by Maximum Likelihood) ver. 4.9d (http://abacus.gene.ucl.ac.uk/software/paml.html; Yang 2007) was also applied for the test.

The sequences of the common ancestors, node 1 (sechellia/simulans) and node 2 (node 1/melanogaster), were estimated, and the substitution history of the ELYS-like domain was reconstructed on the consensus unrooted phylogenetic tree: ((sechellia, simulans), melanogaster), yakuba (Lachaise and Silvain 2004). The ancestral state of node 2 was not determined unambiguously for three sites. We assumed that each replacement substitution took place with an equal probability in three branches (node 2–yakuba, node 2–melanogaster and node 2–node 1). Thus, these were in total calculated as 1/3 × 3 = 1 replacement in each branch.

### Data availability

All *Drosophila* stocks, DNA clones and reagents are available upon request. Viability test for the *Elys* mutations is shown in Table S1. Sperm penetration to the eggs is shown in Table S2. Interaction between *Elys* and *Nup37* is shown in Table S3. The lethal stage of *Elys/Y*; *Df-Nup160/+* males was determined (Table S4). The lethal stage of *Elys/Y*; *Df-Nup96/+* males was determined (Table S5). The cross between *Elys/FM7c*; *Df(2L)Nup160M190/CyO* females and *Elys/Y* males is shown in Table S6. The cross between *Df(3R)/TM6C* females and *D. simulans Lhr* males is shown in Table S6. Sperm were visualized by *dj-GFP* in the eggs from *Elys* mutant females (Figure S1). Mating scheme to determine the lethal stage of *Elys/Y*; *Df-Nup160/+* is shown in Figure S2. Supplemental material available at Figshare: https://doi.org/10.25387/g3.6279446.

## Results

### Description of the Elys mutations

X-linked *CG14215* (X:19,652,305–19,659,407 [+]) of *D. melanogaster* (FlyBase ID FBgn0031052) encodes a protein of 2,111 amino acids (aa) that includes an ELYS-like domain at aa 714–922 (InterPro accession number Q9VWE6; UniProtKB – X2JG50; Finn *et al.* 2017). We recovered two frameshift alleles (*Elys^2^* and *Elys^5^*) that truncate the majority of the coding potential; aa 372 and 367 are predicted to be stop codons, respectively (Figure 1). Surprisingly, the mutants were viable and male-fertile (Supplemental Material, Table S1) but female-sterile in homozygotes (Table 1). Thus, the mutations can be maintained via heterozygous (*Elys/FM*) females and hemizygous (*Elys/Y*) males (or *FM/Y* males), where *FM* (first multiple) stands for a balancer X chromosome; rare *FM* homozygotes are also present in the stocks.

**Table 1:** Hatchability of eggs from females crossed with wild-type (OR) males

	Number of eggs		
Maternal genotype*^a^*	Collected	Hatched	Hatchability, %
*Elys^2^/FM7c*, *Elys^+^* (control)	222	184	82.9
*Elys^5^/FM7c*, *Elys^+^* (control)	208	177	85.1
*Elys^2^/Elys^2^*	204	0	0
*Elys^5^/Elys^5^*	203	0	0
*Elys^2^/Elys^5^*	1,068	0	0
*Elys^2^/Df(1)ED7620*, *Elys^–^*	219	0	0
*Elys^5^/Df(1)ED7620*, *Elys^–^*	209	0	0
*Elys^2^/Df(1)BSC871*, *Elys^–^*	573	0	0
*Elys^5^/Df(1)BSC871*, *Elys^–^*	209	0	0
*Elys^2^/Elys^2^*; *Dp(1;3)DC365*, *Elys^+^/TM6C*	240	220	91.7
*Elys^5^/Elys^5^*; *Dp(1;3)DC365*, *Elys^+^/TM6C*	237	222	93.7

a See text for the full genotype. To obtain *Elys* hemizygotes, *Df(1)ED7620/FM7h* or *Df(1)BSC871/FM7h* females were crossed with *Elys/Y* males. *Elys/Df(1)ED7620* females exhibited etched abdominal tergites.

The homozygous (*Elys/Elys*) and hemizygous (*Elys/Df*) females produced eggs, but the eggs never hatched when crossed with wild-type males (Table 1). Furthermore, the *Elys^+^* transgene on chromosome 3, *Dp(1;3)DC365*, rescued the effect of *Elys* (Table 1); the duplication segment (X:19,624,757–19,716,729; FlyBase ID FBab0046817) carries 22 X-linked protein-coding genes including *Elys* and two ncRNA genes (Venken *et al.* 2010). We can even maintain *Elys*; *Dp(1;3)DC365* as a viable stock. Sperm were observed in the unhatched eggs when visualized by *dj-GFP* (Figure S1 and Table S2). Thus, the *Elys* mutations are recessive female-sterile or maternal-effect lethal.

### Disruption of mitotic progression of the first cleavage division by maternal effects of Elys mutations and Nup160^sim^ introgression

The *Drosophila* embryo remains a syncytium for the first two hours of development, where 13 rounds of nuclear division take place rapidly (Foe and Alberts 1983). To gain insights into the primary effect of the *Elys* mutations on embryonic development, we fixed embryos 10–30 min after deposition and carried out cytological analysis. Our comparative analysis of embryonic progeny produced by *Elys* mutant females (*Elys^2^* or *Elys^5^* homozygotes) and the control females (*Elys^2^* or *Elys^5^* heterozygotes) revealed significant differences in the progression of the earliest cycles. Embryos from females mutant for *Elys* did not display mitotic progression; there was instead the accumulation of characteristics representing the first mitotic cycle (Table 2). Further investigation uncovered the maternal-effect lethality resulting from a terminal arrest in a metaphase-like state of the first cleavage division (Table 3; see below). The phenotype was essentially identical in the two *Elys* mutant strains.

**Table 2:** Development of embryos 10–30 min after deposition

Maternal genotype*^a^*	Number of embryos observed	Stages of embryos: Frequency, %			
		Meiosis or pronuclear stages	Mitosis		
			1^st^ cycle	2^nd^ cycle	3^rd^ cycle and beyond
*Elys^2^/FM7c*, *Elys^+^* (control)	89	1.1	9.0	25.8	64.0
*Elys^5^/FM7c*, *Elys^+^* (control)	67	9.0	10.4	20.9	59.7
*Elys^2^/Elys^2^*	50	4.0	96.0	0	0
*Elys^5^/Elys^5^*	55	1.8	98.2	0	0

a Females were crossed with wild-type (OR) males.

**Table 3:** Mitotic staging in the 1^st^ cleavage division*^a^*

Maternal genotype	Number of embryos observed	Mitotic stages: Frequency, %				Embryos with free asters (%)
		Prophase	Prometaphase–metaphase	Anaphase–telophase	Unidentified	
*Elys/FM7c*, *Elys^+^* (control)*^b^*	15	6.7	40.0	46.7	6.7	0
*Elys^2^/Elys^2^*	48	0	95.8	0	4.2	70.8
*Elys^5^/Elys^5^*	54	0	88.9	0	11.1	79.6

a Embryos are from the 1^st^ cycle column of Table 2.

b The *Elys* mutation is *Elys^2^* or *Elys^5^*.

**Figure 2:**
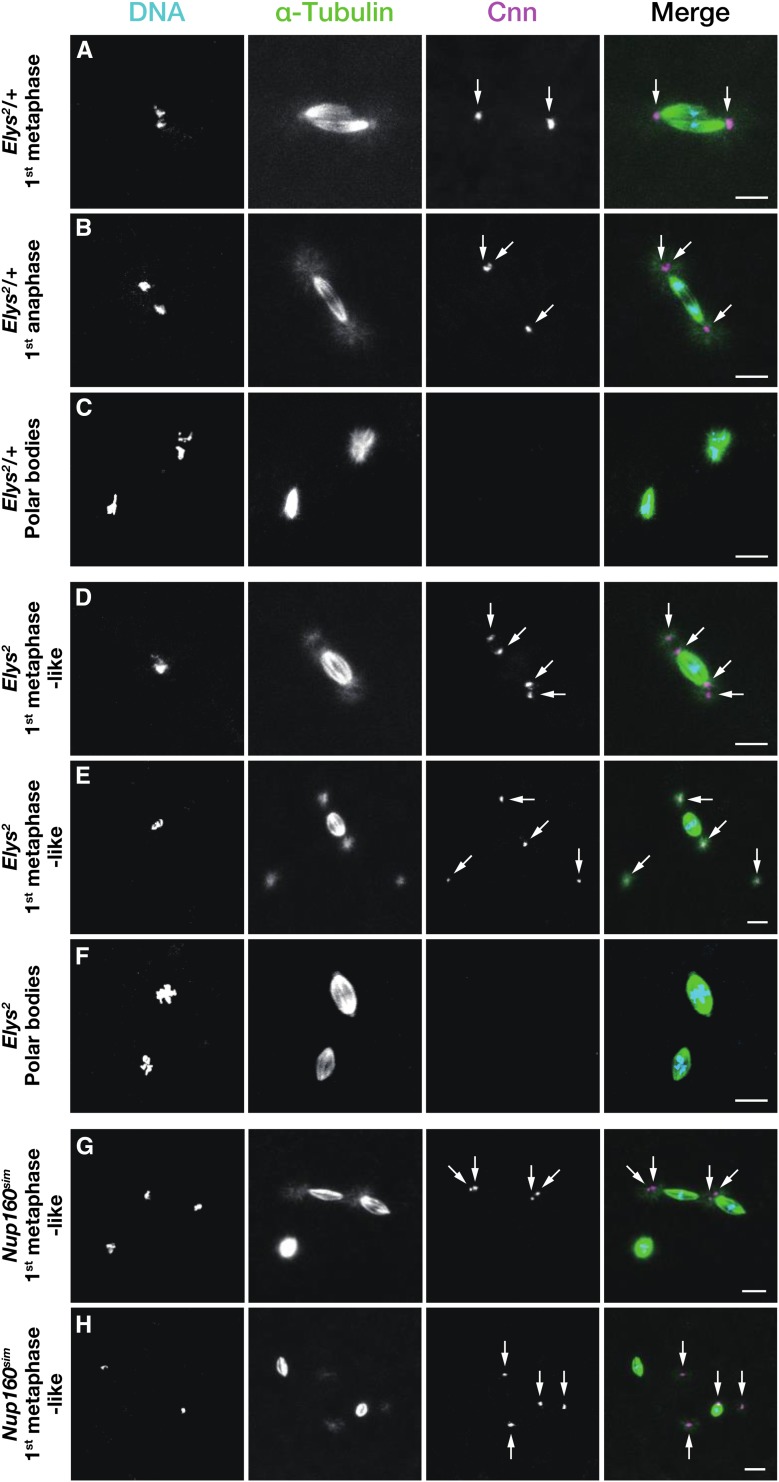
Mitotic arrest phenotypes of embryos produced by *Elys^2^* mutant females and *Int(2L)D+S*, *Nup160^sim^/Df(2L)Nup160M190* females. Embryos fixed in 10–30 min after deposition were treated with antibodies against α-Tubulin (green in merged images) for microtubules and Centrosomin (Cnn, magenta) for centrosomes, as well as the DNA dye DAPI (light blue). (A–C) Embryos of *Elys^2^/+* females were the control. (D–F) Embryos of *Elys^2^* homozygous females, showing developmental arrest at the first cleavage division. (G, H) Embryos of *Nup160^sim^/Df(2L)Nup160M190* females, showing developmental arrest at the first cleavage division. (A) Metaphase of the typical gonomeric mitosis of the first cleavage division. The dual spindle (see text) is organized around the two groups of the chromosomes in juxtaposition. The aster is present at each of the common poles with a single focus of Cnn labeling at each pole. (B) Anaphase of the first cleavage division. Chromosome groups of maternal and paternal origin converge as they synchronously migrate toward the poles and appear as single chromosome masses. The growth of astral microtubules is prominent, and centrosomes are detected as two foci (shown in the upper left pole). (C) Polar bodies with the normal, diffuse or unfocused arrangement of microtubules in the same embryo as (B). (D, E) Note abnormal separation of sister centrosomes around the poles (D) and the individualized centrosomes detaching from the spindle as free asters (E). (F) Polar bodies of the same embryo as in (E). Acentrosomal spindles with a bipolar orientation are often assembled around the chromosomes of polar bodies in embryos from *Elys* mutant females. (G) A bifurcated configuration of the dual spindle. The tandemly oriented two small spindles are connected at the central poles with an aster organized around individualized sister centrosomes. One of the distal poles is astral and the other anastral. A subset of the polar bodies with the normal, circular configuration of microtubules is shown at the lower left. (H) The embryo contains two groups of chromosomes that are distantly located in the cytosol and are encompassed by microtubule arrays of high density. Among four individual centrosomes, three are present as free asters, whereas the remaining one is attached to one of the spindles. Arrows indicate the centrosomes. The scale bars represent 10 μm.

The normal mitosis of the first cleavage division in *Drosophila* is gonomeric (Huettner 1924; Guyénot and Naville 1929; Callaini and Riparbelli 1996; Williams *et al.* 1997; Loppin *et al.* 2015); after DNA replication in nuclei from the ovum and sperm, the haploid complements persist in separate groups on a bipolar spindle composed of two units of microtubule arrays, which we refer to as the dual spindle (Figure 2A). The two units of microtubule arrays share the spindle poles, where the entire set of chromosomes is gathered at telophase. The *Elys* mutations affected the arrangement of the chromosomes and microtubule configurations of the dual spindle, because only spindles that appeared to be composed of a single unit of microtubule arrays with indiscriminately conjugated chromosomes were observed among all 102 embryos obtained from *Elys^2^* and *Elys^5^* females (Figure 2, D and E). In addition, centrosomes behaved in a peculiar manner in the embryos. An analysis of these centrosomes by Cnn immunolabeling showed that, in control embryos, the centrosome is present as a single focus at each of the spindle poles during metaphase of the first cleavage division but then splits into two adjacent foci as early as anaphase (Figure 2, A and B). In embryos of *Elys* mutant females in the first mitotic cycle, however, sister centrosomes were separate, giving rise to two discrete foci even when centrosomes were situated at the pole of the metaphase-like spindle (Figure 2D). Remarkably, individualized centrosomes often detached from the spindle poles and were randomly located in the cytoplasm. We detected free asters with Cnn labeling in >70% of the embryos from both *Elys^2^* and *Elys^5^* females, whereas these were never seen in control embryos (Table 3). We observed up to four free asters within an embryo, indicative of arrest at the first cleavage division. When a spindle pole was devoid of centrosomes, the spindle appeared to be shorter in length and roundish (Figure 2E). It is also noteworthy that, in some embryos from *Elys* mutant females, polar bodies anomalously formed bipolar spindles that lacked centrosomes (Figure 2F; for control see Figure 2C), although their location within the embryo was substantively unaffected, lying near the cortex.

We reported previously that *Nup160^sim^* induces maternal-effect lethality subsequent to sperm penetration in *D. melanogaster* (Sawamura *et al.* 2004), reminiscent of the above-mentioned embryonic phenotype that was due to the *Elys* mutations. Embryos from females hemizygous for *Nup160^sim^* generally arrested their development in a metaphase-like state of the first mitotic cycle (Figure 2, G and H), as is the case with the embryos from *Elys* mutant females. Most (49/50) of the embryos had a total of two to four centrosome foci, whereas the one exception contained eight foci, which might have been attributable to another round of the centrosome cycle or the occurrence of dispermy (insemination by two sperm). Strikingly, *Nup160^sim^* also caused abnormal centrosome behavior, which manifested as free asters in the cytoplasm in ∼75% (38/50) of the embryos. A noticeable difference between the effect of the *Elys* mutations and that of *Nup160^sim^* could be discerned in the deformed mitotic figures that they exhibited. In the embryos of the *Nup160^sim^* females, the union within the dual spindle was partially (12/49, Figure 2G) or thoroughly (24/49, Figure 2H) dissolved, resulting in two distinct spindles, each of a small size. In addition, unlike the *Elys* mutations, *Nup160^sim^* did not affect microtubule configurations of the polar bodies (Figure 2G). Taken together, both the *Elys* mutations and the *Nup160^sim^* introgression commonly affected most, if not all, aspects of the first cleavage division, including mitotic centrosome behavior.

### Synthetic lethality caused by Elys and Nups

Based on the phenotypic similarity between the *Elys* mutations and *Nup160^sim^* introgression, we expected to find a genetic interaction between *Elys* and *Nups*. We thus made double mutants of *D. melanogaster* that carry an *Elys* mutation on the X chromosome and are hemizygous for either of nine autosomal Nup107–160 subcomplex genes. *Elys/FM*; *+/+* females were crossed with *+/Y*; *Df/Bal* males, where *Df* and *Bal* stand for a *Nup* deficiency and a balancer, respectively (Table 4). *Elys/Y*; *Bal/+* males were viable because the balancer contains the wild-type *Nup^+^* (control), but *Elys/Y*; *Df/+* males, which carried only one dose of the *Nup*, were lethal (*Nup96*: viability, 0), semi-lethal (*Nup160*: viability, 0.01–0.04) or had low viability (*Nup37*: viability, 0.13–0.14). It must be stressed here that the lethality caused by the *Elys* mutations or the *Nup160^sim^* introgression is maternal but the synthetic lethality caused by *Elys* and *Nups* double mutants is zygotic. Even in the last case (*Nup37*), most of the *Elys/Y*; *Df/+* males died during or just after emergence: 88.9% (24/27) in *Elys^2^* and 82.9% (29/35) in *Elys^5^*. The lethality of *Elys/Y*; *Df/+* males was confirmed by using additional *Nup37* deficiencies (Table S3; viability, 0.01–0.18). An exception is *Df(3R)ED10946* (viability, 1.02), but we suspect that this deficiency differs from the computational prediction and does not delete *Nup37*; in fact, *Df(3R)ED10946* was viable, although the other deficiencies were lethal, when they were made transheterozygous against *Df(3R)ED10953*. Thus, three of the nine genes (*Nup37*, *Nup96* and *Nup160*) exhibited haploinsufficiency (*e.g.*, hemizygous lethal) in the genetic background of the *Elys* mutations. The lethal stage of the *Elys/Y*; *Df/+* males was late pupal in *Nup160* and *Nup96* (Figure S2, Table S4 and Table S5; for the staging see Bainbridge and Bownes (1981)). We also determined that the lethality of the *Elys* and *Nup* double mutants is not sex-specific. Not only *Elys/Y*; *Df/+* males but also *Elys/Elys*; *Df/+* females were lethal when *Nup160* was made hemizygous (Table S6).

**Table 4:** Interaction between *Elys* and *Nups**^a^*

				Number of offspring														
				Females				Males				Maternal nondisjunctional						
			*Elys* locus	*Elys*/+		+/+		*Elys*/Y		+/Y		*Elys/+/*Y		+/O		Exceptional	Ambiguous*^d^* class III	Viability of *Elys/Y*; *Df/+*
			*Nup* locus	+/+	*Df/+*	+/+	*Df/+*	+/+	*Df/+*	+/+	*Df/+*	+/+	*Df/+*	+/+	*Df/+*	males*^c^*	or	males*^e^*
Locus examined	Paternal genotype*^b^*	*Elys* allele						(class I)	(class II)	(class III)	(class IV)						class IV	(class II)
*Nup98–96*	*Df(3R)BSC489/TM6C*	*2*		144	169	114	130	145	**0**	121	110	1	1	1	2	0	–	**0**
		*5*		221	218	194*^f^*	212	180	**0**	196	194	0	2	5	3	0	–	**0**
*Nup96*	*Nup98–96^339^/TM3*	*2*		167	204	125	144	128	**0**	100	128	1	1	2	3	0	–	**0**
		*5*		221	243	182	196	210	**0**	126	145	2	1	1	3	0	–	**0**
*Nup160*	*Df(2L)Nup160M190/CyO*	*2*		120	115	88	96	101	**3**	86	69	1	2	0	0	0	19	**0.04**
		*5*		189	154	181	152	161	**1**	140	141	3	0	0	0	0	15	**0.01**
*Nup37*	*Df(3R)ED10953/TM6C*	*2*		283	293	222	231	250	**27**	212	163	1	0	4	2	0	–	**0.14**
		*5*		276	303	255	252	286*^h^*	**35**	194	182	2	3	2	0	1	–	**0.13**
*Nup133*	*Df(3R)ED6091/TM6C*	*2*		285	263	221*^g^*	224	251	**157**	199	190*^h^*	1	1	1	2	2	–	**0.66**
		*5*		252	235*^g^*	227	229	197	**154**	214*^h^*	197	1	3	4	3	3	–	**0.85**
*Nup44A*	*Df(2R)Exel6055/CyO*	*2*		268	263	211	199	299	**278**	172	174	4	2	1	3	1	18	**0.92**
		*5*		278	264*^g^*	258	240	269	**194**	189	206	3	2	3	8	0	9	**0.66**
*Nup43*	*Df(3R)ED5815/TM6C*	*2*		264*^f^*	236	238	222	214*^h^*	**112**	190	125	0	2	0	1	0	–	**0.80**
		*5*		317	290	262	266	265*^i^*	**135**	251	154	3	0	1	1	0	–	**0.83**
*Nup107*	*Df(2L)Exel8026/CyO*	*2*		198	198	165	157	170	**179**	125	158	2	0	4	4	0	10	**0.83**
		*5*		191	206	154	149	173	**165**	142	140	1	3	2	3	1	10	**0.97**
*Nup75*	*Df(2R)ED3610/CyO*	*2*		207	223	186	185	237	**225**	164	148	1	2	1	3	0	–	**1.05**
		*5*		253	251	218	226	203	**281**	197	175	1	2	0	2	0	–	**1.56**
*Sec13*	*Df(3R)BSC56/TM6C*	*2*		239	227	215	198	215	**223**	210	133	1	5	2	1	0	–	**1.64**
		*5*		238	244	199	236	230	**237**	193	156	2	2	5	6	0	–	**1.27**

a Males were crossed with *Elys/FM7c* females. The replicates that produced maternal nondisjunctional flies at high frequency were not included in the data, because some of the mothers must have been XXY.

b Full genotypes are available upon request. *Df(3R)ED10953* exhibits a slight Minute phenotype, because the *Nup37* locus is close to *RpS27*.

c Presumably produced by the break in *FM7c* (see Hutter 1990).

d The presence of the chromosome 2 balancer could not be determined.

e Calculated as (class II × class III)/(class I × class IV).

f One was Minute, presumably haplo-4.

g One was a gynandromorph.

h One was apparently paternal nondisjunctional XO.

i Two were apparently paternal nondisjunctional XO.

### Not only Nup96 and Nup160 but also Nup37 may cause hybrid lethality

In the cross between *D. melanogaster* females and *D. simulans* males, hybrid males are lethal but are rescued by the *Lhr* (*Lethal hybrid rescue*) mutation of *D. simulans* (Sturtevant 1920; Watanabe 1979). When *Nup96^sim^* or *Nup160^sim^* is made hemizygous by a deficiency chromosome of *D. melanogaster* or made homozygous by an introgression from *D. simulans*, the hybrid males cannot be rescued by *D. simulans Lhr* (Presgraves *et al.* 2003; Tang and Presgraves 2009; Sawamura *et al.* 2010). This is because *Nup96^sim^* and *Nup160^sim^* behave as recessive hybrid incompatibility genes (Strategy 2 of Sawamura 2016). In other words, a gene or genes from *D. melanogaster* (incompatibility partner) result in hybrid inviability in the genetic background of *Nup96^sim^* or *Nup160^sim^* homozygote (or hemizygote).

We reported above that not only *Nup96* and *Nup160* but also *Nup37* exhibited haploinsufficiency in the genetic background of the *Elys* mutations. This raises the possibility that *Nup37* is also a gene for hybrid incompatibility. We thus made crosses by using deficiency chromosomes that lack *Nup37*. The interspecific crosses were very difficult, presumably because the deficiencies affect mating behavior; the hemizygotes exhibited the Minute phenotype resulting from the haploinsufficiency of closely linked *RpS27* (*Ribosome protein S27*; Marygold *et al.* 2007). Crossing was successful only when *Df(3R)ED10953* was used, and the male hybrids hemizygous for *Nup37^sim^* were not rescued by *Lhr* (Table S7), although we cannot rule out the possibility that the lethality is a secondary effect of *RpS27*. Thus, not only *Nup96* and *Nup160* but also *Nup37* may be hybrid incompatibility genes.

### Adaptive evolution of Elys in Drosophila

**Figure 3:**
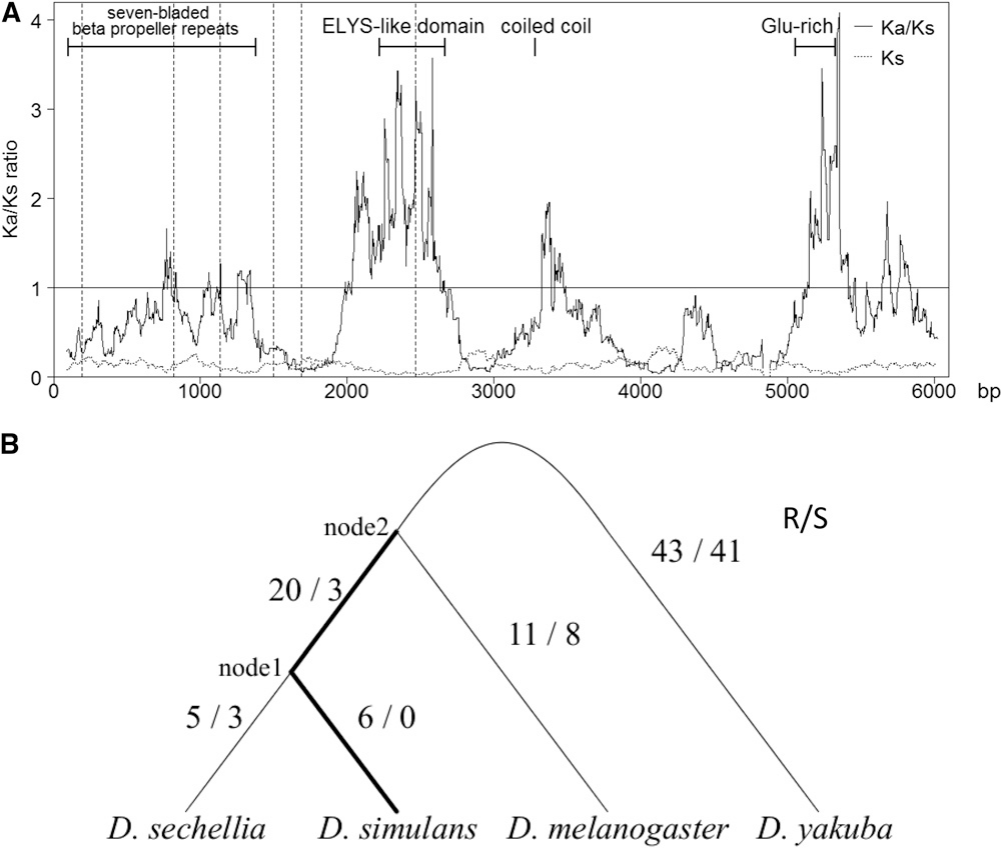
A comparison of *Elys* gene sequences among *Drosophila* species. (A) *K_a_*/*K_s_* test (180-bp sliding widow) between *D. melanogaster* and *D. simulans* (exons are separated by vertical dashed lines). The horizontal line (*K_a_*/*K_s_* = 1) indicates neutral evolution. (B) Replacement (R) *vs.* synonymous (S) substitutions in the ELYS-like domain.

Hybrid incompatibility genes generally evolve rapidly (Ting *et al.* 1998; Barbash *et al.*, 2003; Presgraves *et al.* 2003; Brideau *et al.* 2006; Tang and Presgraves 2009). We thus compared the *Elys* gene sequences of *D. melanogaster* and *D. simulans*. Although *K_a_*/*K_s_* = 0.53 when the entire coding sequence was used, the sliding window analysis indicated positive natural selection (*K_a_*/*K_s_* > 1) around the ELYS-like domain and the Glu-rich domain of the gene (Figure 3A). In fact, *K_a_*/*K_s_* = 1.51 and 1.10 for these two domains, respectively, even though the Glu-rich domain is 49 aa shorter in *D. simulans*. The sequences of *D. yakuba* and *D. sechellia* were added to the comparison of the ELYS-like domain, and amino acid replacements and synonymous substitutions were counted in each branch of the phylogenetic tree (Figure 3B). Positive natural selection seems to have occurred on the route from node 2 (the common ancestor of *D. melanogaster* and *D. simulans*) to *D. simulans*, as indicated by the 26 replacements *vs.*. 3 synonymous substitutions. This was confirmed by the branch model of PAML; not significant for the full-length *Elys* sequences but significant for the ELYS-like domain (*P* = 0.008 for the *D. simulans* branch after sprit from *D. melanogaster* and *P* = 0.048 for *D. simulans* branch after the sprit from *D. sechellia*).

## Discussion

### ELYS function in D. melanogaster

ELYS plays an important role in the NPC assembly, as noted above. Therefore, it was a surprise that *Elys* is dispensable for viability and male fertility in *D. melanogaster* (Figure 1 and Table S1). *D. melanogaster* might have another gene or genes, the function of which is redundant with *Elys*, although we have not found genes with sequence similarity. Similar to mutations in the *C. elegans* homolog, *mel-28* (Fernandez *et al.* 2014; Gómez-Saldivar *et al.* 2016), *D. melanogaster Elys* exhibited a maternal effect (Table 1). Females mutant for the gene produced apparently normal eggs in which sperm can penetrate (Figure S1 and Table S2), but the development of the resulting embryos never progressed beyond the first mitotic division (Figure 2 and Table 2).

In the present study, we carefully examined the maternal effect of the *Elys* mutations (Table 3) and *Nup160^sim^* introgression in early *Drosophila* embryos and showed that they share the embryonic phenotype of developmental arrest in a metaphase-like state of the first cleavage division. Therefore, the *Nup160^sim^* introgression in *D. melanogaster* appears to behave like a loss-of-function allele of *Elys*. The prior steps of fertilization, such as the establishment of the sperm aster and pronuclear apposition, were unaffected, and no figures showing anaphase of the first cleavage division or later were observed. In these embryos, abnormally individualized centrosomes and their dissociation from the spindle poles were obvious, implying ELYS and Nup160 in mitotic centrosome behavior. Consistently, a proteomic analysis of *Drosophila* embryonic centrosomes shows that ELYS is actually a centrosome component (see Table S1 of Müller *et al.* 2010), although its function has not yet been established. Centrosomal localization of Nup160 is unknown in *Drosophila*, but the protein has been detected in spindle poles and proximal spindle fibers of HeLa cells (Orjalo *et al.* 2006).

The developmental arrest could be accounted for by failure in structural changes of the nuclear envelope during the semi-open mitosis of early *Drosophila* embryos and/or disrupted interactions between the kinetochore and microtubules (Güttinger *et al.* 2009). Both ELYS and the Nup107–160 subcomplex can be detected in an interdependent manner at spindle poles and kinetochores (Zierhut and Funabiki 2015). Also, the halting of mitotic progression could reflect the abnormal persistence of spindle-associated Cyclin B owing primarily to the dissociation of centrosomes from spindle poles, as the polar localization of centrosomes is required to initiate local destruction of Cyclin B in mitotic spindles of the *Drosophila* syncytium (Huang and Raff 1999; Wakefield *et al.* 2000). The fact that the *Elys* mutations and the *Nup160^sim^* introgression result in very different outcomes with respect to the deformed morphology of the first mitotic spindle suggests that the ELYS and Nup160 proteins may have both common and distinct roles in the spindle assembly characteristic of the first cleavage division.

The present cytological study clearly demonstrates that ELYS and Nup160 are commonly involved, at a minimum, in centrosome behavior during the first cleavage division. Studies on subcellular localization of the ELYS and Nup160 proteins and their protein-protein interactions are needed to further elucidate their functions.

Because ELYS determines the subcellular localization of the Nup107–160 subcomplex (Belgareh *et al.* 2001; Boehmer *et al.* 2003; Harel *et al.* 2003; Walther *et al.* 2003; Loïodice *et al.* 2004; Franz *et al.* 2007; Gillespie *et al.* 2007; Rasala *et al.* 2006, 2008; Doucet *et al.* 2010; Bilokapic and Schwartz 2013; Inoue and Zhang 2014), we expected genetic interaction between *Elys* and *Nups*. Among the nine Nup107–160 subcomplex components examined, *Nup37*, *Nup96* and *Nup160* indeed exhibited haploinsufficiency in the genetic background of the *Elys* mutations (Table 4, Table S3 and Table S6); *Elys/Y*; *Df/+* males were lethal at the pupal stage (Figure S2, Table S4 and Table S5). Interestingly, those three Nups are located in close proximity in the NPC (see Figure 1 of Hurt and Beck 2015). Furthermore, Bilokapic and Schwartz (2012) have suggested that ELYS binds near an interface of the subcomplex consisting of Nup120 (the yeast homolog of Nup160) and Nup37 in *Schizosaccharomyces pombe*. This might cause the epistatic interaction detected in the present analysis. Notably, the effect of *Elys* mutations and *Nup160^sim^* introgression is different than that of double mutations of *Elys* and *Nups*; the former survived to adulthood on their own and the lethality was only revealed as maternal effect while the latter exhibited a strong zygotic phenotype. These results suggest that ELYS and Nups may act at the same component of the mitotic machinery, or at another unidentified biological process, resulting in more severe synthetic lethal interactions.

Although ELYS sequences are well conserved in metazoans (Rasala *et al.* 2006), our present analysis detected positive natural selection in the ELYS-like domain of the protein in the branch leading to *D. simulans* (Figure 3 and Table S8). This might be the consequence of coevolution between ELYS and Nups. Indeed, recurrent adaptive evolution has been detected in five Nup107–160 subcomplex components (Nup75, Nup96, Nup107, Nup133 and Nup160) and two mobile Nups (Nup98 and Nup153) in *D. melanogaster* and *D. simulans* (Presgraves *et al.* 2003; Presgraves and Stephan 2007; Tang and Presgraves 2009).

### Possible involvement of ELYS in reproductive isolation

Several genes responsible for hybrid lethality between *D. melanogaster* and *D. simulans* have been identified (for recent reviews, see Sawamura 2016; Castillo and Barbash 2017). *Lhr* and *Hmr* (*Hybrid male rescue*), which encode chromatin binding proteins, are one such incompatibility pair (Watanabe 1979; Hutter and Ashburner 1987; Barbash *et al.* 2003; Brideau *et al.* 2006; Thomae *et al.* 2013; Blum *et al.* 2017), and *gfzf* (*GST-containing FLYWCH zinc-finger protein*) is an upstream gene in this incompatibility (Phadnis *et al.* 2015).

*Nup96* and *Nup160* are also involved in reproductive isolation (Presgraves *et al.* 2003; Tang and Presgraves 2009; Sawamura *et al.* 2010). *Nup96^sim^* and *Nup160^sim^* synergistically cause hybrid incompatibility (Sawamura *et al.* 2014), but the *D. melanogaster* alleles of *Nup160* and *Nup96* are not the dominant autosomal incompatibility partner of *Nup96^sim^* and *Nup160^sim^*, respectively (Tang and Presgraves 2015). Then, what is (are) the incompatibility partner(s) of *Nup96^sim^* and *Nup160^sim^*? One can envision that at least one recessive gene must be located on the X chromosome of *D. melanogaster* (X_mel_), because the hybrid inviability is revealed in X_mel_Y_sim_ but not in X_mel_X_sim_, where Y_sim_ and X_sim_ stand for the Y and X chromosomes of *D. simulans*, respectively (Strategy 2 of Sawamura 2016). We here propose that the X-linked *Elys* of *D. melanogaster* may be the incompatibility partner of *Nup96^sim^* and *Nup160^sim^*.

Our proposal is based on three observations. (1) *Elys* mutations mimic the maternal *Nup160^sim^* introgression phenotype in *D. melanogaster* (Figure 2), which suggests that *Elys* affects the same cascade as the *Nup160^sim^* incompatibility. (2) Epistatic interaction was detected between *Elys* and *Nup37*, *Nup96* or *Nup160* in *D. melanogaster* (Table 4). (3) Male hybrids between *D. melanogaster* and *D. simulans* cannot be rescued by the *Lhr* mutation if *Nup37*, *Nup96* or *Nup160* of *D. melanogaster* is deficient (Table S7; Presgraves *et al.* 2003; Tang and Presgraves 2009; Sawamura *et al.* 2010).

In this model we presume that *D. melanogaster* ELYS does not function properly—and thus NPC formation and mitotic centrosome behavior are compromised—if Nup37, Nup96 or Nup160 is from *D. simulans*. We must also note that the incompatible *D. simulans* allele of the Nup107–160 subcomplex genes is recessive; the presence of the *D. melanogaster* allele is enough to avoid incompatibility. Thus, hemizygous *Nup160^sim^* introgression causes female sterility (maternal-effect lethality) with a phenotype that is similar to the *Elys* mutations of *D. melanogaster* (Figure 2). But *Nup96^sim^* introgression does not cause female sterility (Sawamura *et al.* 2014) and *Nup37^sim^* has not been tested.

Recently, *rhi* (*rhino*) and *del* (*deadlock*), which encode piRNA pathway proteins, were shown to be another incompatibility pair (Parhad *et al.* 2017). This pathway might have been adapted to suppress the species-specific transposable element mobilization (Kelleher *et al.* 2012; Parhad *et al.* 2017). ELYS plays an important role in the piRNA pathway; PIWI is released from messenger ribonucleoprotein particles by binding to NPCs via Xmas-2, ELYS and other NPC components (Ilyin *et al.* 2017). The piRNA pathway evolution might result in the incompatibility between *Elys* and *Nups*.

Thus, *Elys* is a candidate for a gene of reproductive isolation between *D. melanogaster* and *D. simulans*, but direct evidence is necessary. We are going to test the viability and female fertility of flies (*D. melanogaster* or the *D. melanogaster*/*D. simulans* hybrid) that carry various combinations of *Elys* and *Nup* alleles.
